# Impact of Anastomotic Leak on Long-Term Survival After Gastrectomy: Results from an Individual Patient Data Meta-Analysis

**DOI:** 10.3390/cancers17152471

**Published:** 2025-07-25

**Authors:** Matteo Calì, Davide Bona, Sara De Bernardi, Yoo Min Kim, Ping Li, Emad Aljohani, Giulia Bonavina, Gianluca Bonitta, Quan Wang, Antonio Biondi, Luigi Bonavina, Alberto Aiolfi

**Affiliations:** 1 IRCCS Ospedale Galeazzi—Sant’Ambrogio, Division of General Surgery, Department of Biomedical Science for Health, University of Milan, 20151 Milan, Italy; matteocali94@gmail.com (M.C.); saradebe.99@gmail.com (S.D.B.); bbonit@icloud.com (G.B.); 2 Severance Hospital—Division of General Surgery, Department of Upper Gastrointestinal Surgery, Yonsei University, Seoul 03722, Republic of Korea; ymkim@yuhs.ac; 3 Department of Gastric Surgery, Fujian Medical University Union Hospital, Fuzhou 350001, China; li.ping@gmail.com; 4 College of Medicine, Prince Sattam bin Abdulaziz University, Alkharj 16273, Saudi Arabia; e.aljohani@psau.edu.sa; 5 Department of Obstetrics and Gynecology, IRCCS MultiMedica, 20138 Milan, Italy; bonavinagiulia@gmail.com; 6 IRCCS Policlinico San Donato, Division of General and Foregut Surgery, Department of Biomedical Sciences for Health, University of Milan, 20122 Milan, Italy; 7 G. Rodolico Hospital, Surgical Division, Department of General Surgery and Medical Surgical Specialties, University of Catania, 95131 Catania, Italy; abiondi@unict.it

**Keywords:** gastric cancer, gastrectomy, anastomotic, leak, survival

## Abstract

Background: Anastomotic leak (AL) is a serious complication after gastrectomy. It is associated with prolonged hospital stay, greater expenses, and increased risk for 90-day mortality. Currently, there is no consensus regarding the effect of AL on OS in patients with GC undergoing gastrectomy. This study was designed to investigate the effect of AL on long-term survival after gastrectomy for gastric cancer. Methods: PubMed, Embase, Scopus, Google Scholar, and Cochrane Library were queried during the search process. The literature search started in January 2025 and was updated in May 2025. The studies analyzed the impact of AL on long-term survival, with the primary outcome being long-term overall survival. Pooled effect size measures included restricted mean survival time difference (RMSTD), hazard ratio (HR), and 95% confidence intervals (CIs). Results: Ten studies (11,862 patients) were included. Overall, 338 (2.9%) patients experienced AL. The RMSTD analysis indicates that at 12, 24, 36, 48, and 60 months, patients with AL tend to live 1.1, 3.1, 5.2, 8.1, and 10.6 months shorter, respectively, compared to those who did not develop AL. All results were statistically significant with *p* < 0.0001. The time-dependent HRs analysis for AL versus no AL shows a higher mortality hazard in patients with AL at 12 (HR 1.32, 95% CI 1.11–1.58), 24 (HR 1.61, 95% CI 1.34–1.92), 36 (HR 1.55, 95% CI 1.27–1.91), 48 months (HR 1.22, 95% CI 1.02–1.53), and 60 months (HR 0.79, 95% CI 0.59–1.10). Conclusions: This research appears to indicate a clinical impact of AL on long-term OS after gastrectomy. Patients experiencing AL appear to have an increased risk of mortality within the initial four years of follow-up.

## Simple Summary

Anastomotic leak (AL) is a relevant complication after gastrectomy. This study aimed to assess the effect of AL on long-term survival following gastrectomy for gastric cancer (GC). A total of ten studies, encompassing 11,862 patients, were included. Among them, 338 (2.9%) experienced AL. The RMSTD analysis indicates that at 12, 24, 36, 48, and 60 months, patients with AL tend to live 1.1, 3.1, 5.2, 8.1, and 10.6 months shorter, respectively, compared to those who did not develop AL. These findings seem to suggest that AL has a significant clinical impact on long-term overall survival (OS) after gastrectomy, with affected patients facing a heightened mortality risk during the first four years of follow-up.

## 1. Introduction

According to the Global Cancer Statistics (GLOBOCAN) 2022, gastric cancer (GC) is the fifth most common and most deadly tumor worldwide, thus representing a major global health challenge [1]. The incidence varies geographically across the globe, with the highest incidence in Asia, where approximately 70% of new cases and deaths occur [2]. Radical surgery with lymphadenectomy remains the primary treatment for resectable gastric cancer. Despite significant advances in surgical techniques, subtotal and total gastrectomy are still associated with considerable postoperative morbidity, with complication and mortality rates ranging from 11 to 46% and 3 to 20%, respectively [3,4,5,6,7,8,9,10]. Among postoperative complications, anastomotic leak (AL) is the most feared, with a reported incidence ranging from 1.2 to 6.7% [11,12]. AL has an impact on postoperative mortality, length of hospital stays, hospitalization costs, and patients’ quality of life.

Previous studies have investigated the role of postoperative complications on overall survival (OS), but few have specifically focused on AL [13]. Currently, there is no consensus regarding the effect of AL on OS in patients with GC undergoing gastrectomy. While some studies reported that postoperative AL is associated with shorter OS [11,14,15], others have found no significant impact on survival [16].

In this individual patient data (IPD) meta-analysis, we aimed to assess the impact of anastomotic leak on long-term OS in patients with gastric cancer undergoing gastrectomy.

## 2. Materials and Methods

A systematic review was performed according to the Preferred Reporting Items for Systematic Reviews and Meta-Analyses (PRISMA 2020) guidelines [17], without requiring ethical approval. Multiple databases, including PubMed, Embase, Scopus, Google Scholar, and Cochrane Library, were queried. The literature search started in January 2025, was updated in March 2025, and was completed on 15 May 2025. The following search strategy, including MeSH (Medical Subject Headings) terms, was used: (cancer OR carcinoma) AND (stomach OR gastric) AND (anastomotic leak) OR (anastomosis leak) OR (anastomotic leakage) OR (anastomosis leakage) OR (anastomotic failure) OR (anastomosis failure) OR (anastomotic breakdown) OR (anastomosis breakdown) OR (dehiscence) OR (fistula) AND (survival). The complete literature strategy is reported in Appendix A. All titles were examined, relevant abstracts were retrieved, and the reference lists of selected articles were independently evaluated by two authors (M.C., S.D.B.). The systematic review has been registered with the PROSPERO database (CRD420251038636).

### 2.1. Eligibility Criteria

Inclusion criteria: (a) studies evaluating the impact of anastomotic leak on long-term survival after total or distal gastrectomy; (b) articles reporting long-term overall survival (OS) Kaplan–Meier curves; and (c) when several articles originated by the same institution, study group, or dataset, those with the longest follow-up period or largest sample size were selected for inclusion.

Exclusion criteria: (a) studies not written in English; (b) studies lacking a comparative analysis between patients with and without AL following gastrectomy; (c) studies reporting aggregated data for both esophageal and gastric cancers; (d) studies not reporting long-term survival data or Kaplan–Meier curves; (e) studies in which >20% of cases were proximal gastrectomies or gastrectomies performed for purposes other than cancer (e.g., prophylactic gastrectomy); and (f) studies in which >20% of leaks occurred at non-anastomotic sites (e.g., duodenal stump).

### 2.2. Data Extraction

The following data were retrieved from the selected articles: authors, year of publication, country, study design, number of patients, sex, age, body mass index (BMI), American Society of Anesthesiologists (ASA) physical status, tumor characteristics (e.g., histology, TNM classification, pathological stage), tumor location, surgical procedure (e.g., total, distal, proximal gastrectomy) and approach (e.g., open, laparoscopic, robotic, etc.), use of neoadjuvant and/or adjuvant therapy, duration of follow-up, postoperative outcomes and complications, and OS. Three authors (M.C., S.D.B., A.A.) independently gathered all data, which were reconciled at the end of the evaluation process. A fourth author (D.B.) reviewed the database to solve any discrepancies and ensure accuracy.

### 2.3. Outcomes of Interest and Definitions

The primary outcome was OS, defined as the time elapsed from surgery to the last known follow-up or death. Individual patient survival data were derived from Kaplan–Meier curves reported in the included studies or in the related Supplementary Materials. Gastric cancer was defined as any primary histopathologically confirmed neoplasm located at any level of the stomach, including gastroesophageal junction (GEJ) Siewert type III tumors. Anastomotic leakage was defined as any clinical or radiological evidence of dehiscence of the anastomosis. Clinical signs and symptoms suggestive of leakage included output of gastrointestinal content or saliva through a drain, persistent fever, ileus, or symptoms of peritonitis. Leakage was typically confirmed by abdominal computed tomography (CT), contrast swallow studies, or upper endoscopy.

### 2.4. Quality Assessment and Assessment of Certainty of Evidence

Two authors (M.C., S.D.B.) individually assessed the methodological quality of included papers. The ROBINS-I tool version 2 was applied to assess the methodological quality of selected studies [18]. Confounding, classification, selection, intervention, missing data, outcomes measurement, and reporting bias were reviewed. Each domain was assigned “low”, “moderate”, “serious”, or “critical” risk of bias. After completing all seven bias domains, an overall judgement was made. The results were synthesized using the robvis tool (Supplementary Figure S1).

### 2.5. Statistical Analysis

The systematic review findings were qualitatively summarized for a Frequentist meta-analysis of the difference in restricted mean survival time (RMSTD) [19]. The individual patient time-to-event data were reconstructed from Kaplan–Meier curves [20] using Get Data Graph Digitizer software version 5.0 (https://automeris.io/; accessed on 25 March 2025). The pooled RMSTD estimation was conducted using a random-effects multivariate meta-analysis method, accounting for within-trial covariance across different time points. Additionally, a flexible hazard-based regression model was developed using IPD, featuring a normally distributed random intercept. Within periocular analyses, the baseline hazard was characterized by an exponential B-spline of degree 3 without interior knots, selected based on the Akaike information criterion (AIC). The dynamic effects of surgical treatment were modeled as interaction terms between the surgical intervention and the baseline hazard, assessed using a likelihood ratio test. Hazard function plots were synthesized using marginal prediction [21], with statistical significance defined as two-sided *p*-values below 0.05 and 95% confidence intervals calculated. The statistical analysis was performed using R software version 3.2.2 from the R Foundation in Vienna, Austria [22].

## 3. Results

### 3.1. Systematic Review

**Figure 1:**
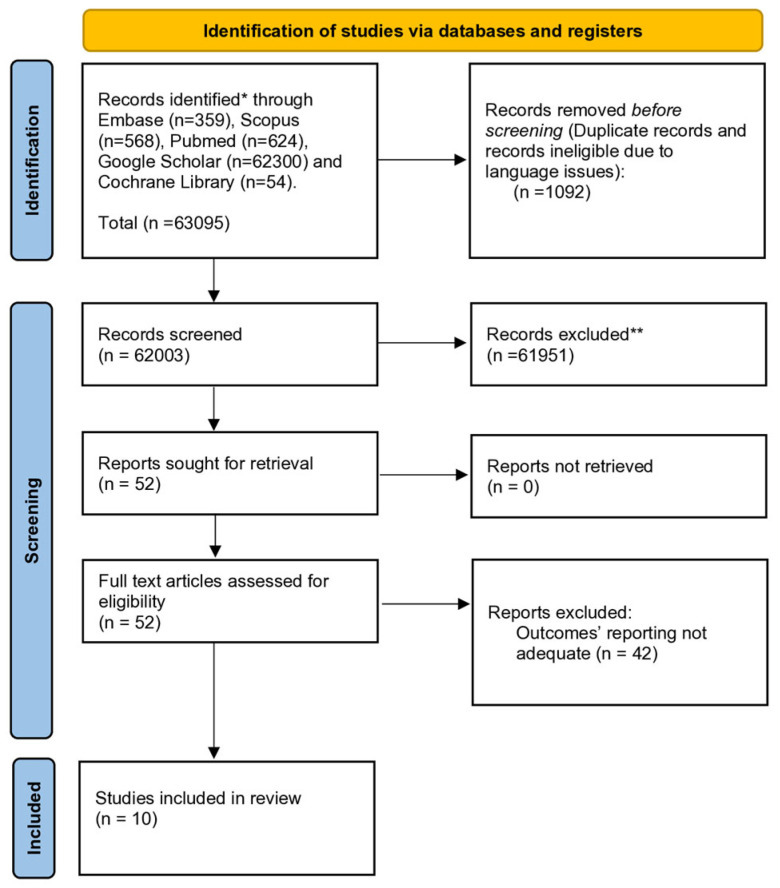
The preferred reporting items for systematic reviews and meta-analyses (PRISMA) diagram. * Records searched with Embase, Scopus, PubMed, Google scholar, and Cochrane library. ** Records excluded by title and abstract screening due to publication type (publications such as conference abstracts, book chapters, and conference posters were excluded) and unrelated topics.

The selection process flowchart is shown in Figure 1. Overall, 62,003 publications were screened after duplicate removal, and 52 were identified for the full-text review. After evaluation, 10 studies [12,15,23,24,25,26,27,28,29,30] met the inclusion/exclusion criteria and were incorporated in the quantitative analysis. All papers were single-center studies except one study that was multicenter [15]. The quality of studies is depicted in Table 1. Overall, 11,862 patients with gastric cancer undergoing gastrectomy were included for quantitative analysis. Of those, 338 (2.9%) experienced AL. The age of the patients ranged between 25 and 94 years, and the majority were males (69.5%). The preoperative body mass index (BMI) and ASA were both reported in five studies, with a majority of patients having a BMI < 25 (52%), and 9.6% of patients having an ASA score ≥ 3. Histology was reported inconsistently across studies [23,24,26,27,29]. Tumors were distributed in the upper (21.3%), middle (23.2%), and lower (34%) stomach. Pathological tumor staging according to the American Joint Committee on Cancer (2002 [15,23], 2010 [25,26]) or Japanese 15th [27] or JGCA1998 [24] or UICC [28] was specified in seven studies (6206 patients): stage 0–I: 32.2%; stage II: 14.2%; stage III: 21.6%; and stage IV: 3.2%. Neoadjuvant treatment, according to different protocols and regimens, was completed in 3.8% of patients, while adjuvant treatment was completed in 7%. Distal (47.5%) and total (32.9%) gastrectomy were the two most commonly reported surgical procedures. Open (48.4%), laparoscopic-assisted (49.5%), and totally minimally invasive (0.1%) approaches were described, while the extension of lymph node dissection (D1/D1+/D2/D3) varied across the studies.

### 3.2. Meta-Analysis—Overall Survival

**Figure 2:**
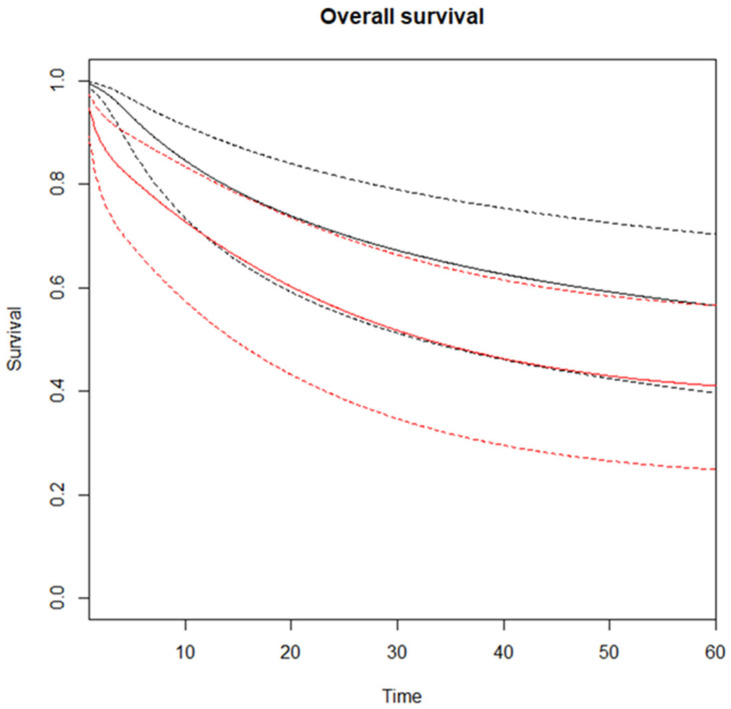
Estimated pooled overall survival (OS) on Y-axis, for noAL (black line) and AL (red line) with time expressed in months on the X-axis. Solid lines represent survival curves, while dotted lines indicate 95% confidence intervals.

**Figure 3:**
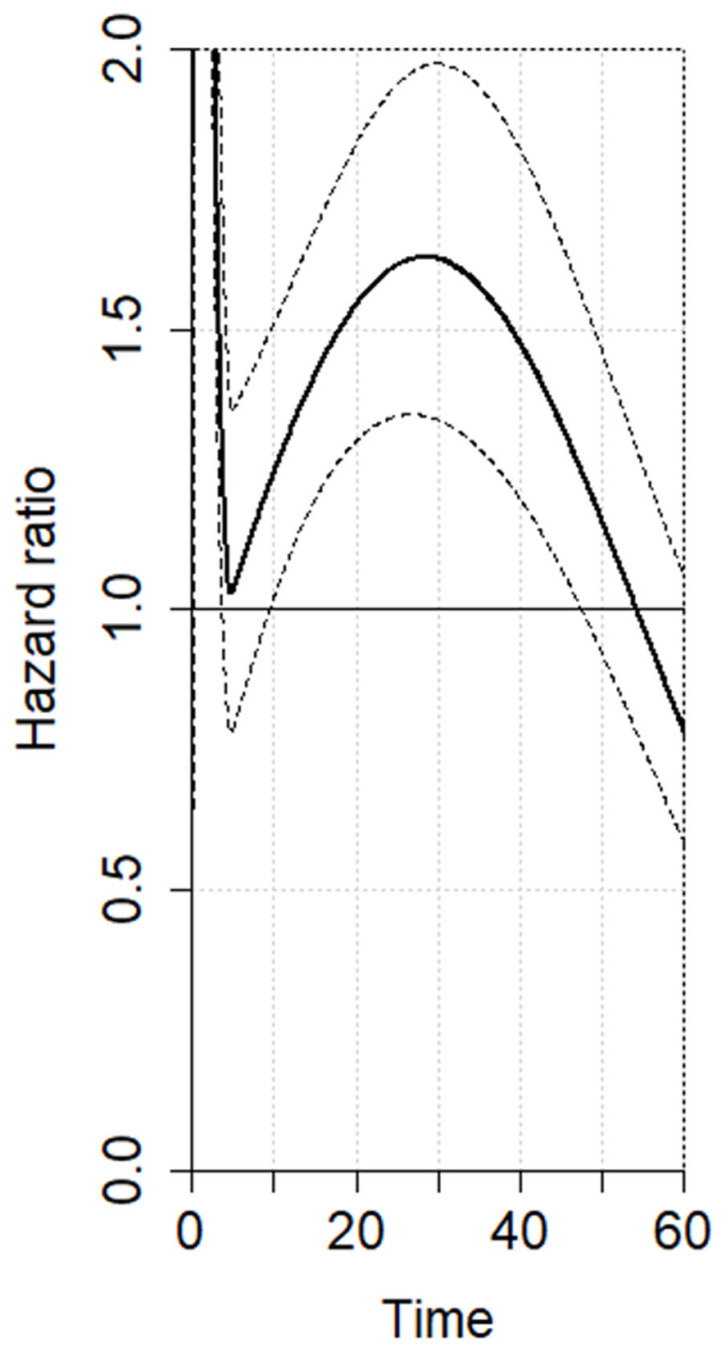
Variation in overall survival hazard ratio over time for AL. The Y-axis represents hazard ratio, while the X-axis denotes time in months. Solid lines illustrate estimated pooled hazards while dotted tracts represent the 95% confidence interval (95% CI).

The evaluation of the RMSTD was conducted on studies reporting Kaplan–Meier curves for overall survival (OS) (n = 10). The RMSTD and the time horizons are detailed in Table 2. Specifically, at 60-month follow-up, the multivariate meta-analysis with analytically derived covariance gives a combined RMSTD estimate of 10.6 months (95% CI 5.7–15.4 months), indicating that patients who did experience AL lived, on average, 10.6 months shorter than those not affected by AL. This result is statistically significant (*p* < 0.0001). The estimated pooled OS for high-volume and low-volume centers is illustrated in Figure 2. Considering the non-proportional hazard model (*p* < 0.001), the time-varying hazard ratios for AL vs. no AL are depicted in Figure 3. AL is linked with a significantly higher hazard for mortality at 12 months (HR 1.32, 95% CI 1.11–1.58), 24 months (HR 1.61, 95% CI 1.34–1.92), 36 months (HR 1.55, 95% CI 1.27–1.91), and 48 months (HR 1.22, 95% CI 1.02–1.53) compared to patients without AL (Table 3). The analysis revealed low between-study heterogeneity (I^2^ = 13%); however, the broad 95% confidence interval (0–62.3%) indicates substantial uncertainty around this estimate, warranting cautious interpretation.

## 4. Discussion

This study seems to suggest a clinical impact of AL on long-term OS after gastrectomy. The RMSTD analysis indicates that patients who did experience AL had an average survival disadvantage of 10.6 months over the 60-month follow-up period. Further, the time-dependent HR analysis shows that patients experiencing AL seem to have a significantly higher mortality risk within up to 48 months compared with patients with no AL.

Recent improvements in long-term survival after gastrectomy may be attributable to advancements in surgical technique [31,32,33], precise lymph node dissection [34,35], improved intra/perioperative management, and new perioperative treatments like immunotherapy. Gastric resection is associated with several potential complications, with AL reported incidence rates of 5.8–6.7% for open and 3.3–4.1% for laparoscopic gastrectomy [36]. AL has been associated with a notable rise in postoperative morbidity, hospital length of stay, increased healthcare costs, and 90-day mortality rates [37]. However, its impact on long-term survival is still debated. In this study, we found that AL after gastrectomy seems to have a significant clinical impact on long-term OS. The RMSTD estimation specifically found that postoperative AL negatively impacts OS, with patients experiencing AL surviving, on average, 10.6 months shorter than those who did not experience AL over the 60-month follow-up period. This is similar to what has been reported by Yoo et al. [23]: they conveyed a significantly reduced overall mean survival in patients who experienced postoperative AL compared to those without AL (30.5 vs. 96.2 months; *p* < 0.001). Notably, AL was an independent predictor for higher mortality in the logistic regression analysis (HR: 3.58, 95% CI: 2.29–5.59) [23]. Similarly, Kamarajah et al. [14], in their retrospective study with 969 patients, indicated a negative impact of AL on long-term overall survival (HR 3.96; *p* < 0.001). In the same way, Andreou et al. [38] observed a negative impact of AL on survival (HR 1.74; *p* = 0.037). In contrast, Saunder and colleagues in their retrospective analysis, including 1100 patients, concluded that, beyond the acute postoperative period, anastomotic leak did not adversely affect long-term survival [16]. Similarly, Climent et al. in their retrospective single-center study with 271 patients concluded that, among postoperative complications Clavien–Dindo (CD) ≥ II, sepsis and intra-abdominal sepsis were not associated with a negative effect on the oncologic outcome after curative gastric cancer resection [39]. Various hypotheses have been suggested to explain the impact of AL on survival, but the exact mechanism remains complex. The onset of AL may deteriorate the general health of patients and impede or delay their access to adjuvant treatment. Additionally, other factors may contribute to this negative outcome. One possibility is that AL is more prevalent in patients with poorer disease biology, particularly those with more aggressive tumors that have a higher likelihood of recurrence. Another concerning possibility is that AL itself may affect long-term prognosis. It has been proposed that AL may facilitate the release of cells from the gut lumen, potentially leading to local recurrence, with perivisceral collections acting as a site for dissemination. However, this hypothesis has primarily been suggested for other gastrointestinal tumors, such as esophageal and rectal cancers [40,41], while studies on the effects of AL in patients undergoing subtotal or total gastrectomy for gastric cancer are limited. The most plausible hypothesis is that the leak may trigger an immunosuppressive response that promotes disease recurrence in two ways: First, the inflammatory response to the leakage could elevate levels of mediators like tumor necrosis factor α, interleukin 1β, and interleukin 6, which may encourage proliferation, cell motility, invasiveness, and survival of circulating cancer cells [42]. Second, the immune response against cancer may be less effective during the period of leakage [43].

Following the RMSTD analysis, we evaluated survival data and hazard ratios (HRs) derived from the survival curves. HRs are commonly used to estimate treatment effects for time-to-event outcomes, reflecting the hazard rate ratio between experimental and control groups over the study period. However, HRs fluctuate over time, offering insight into both the magnitude and direction of survival outcomes. The analysis of risk-time variations indicates that mortality risk varies over time. The HR curve initially peaks at HR = 2, associated with increased postoperative mortality at 90 days. It then shows a gradual decline that remains statistically significant up to 48-month follow-up. At that point, the lower bound of the 95% confidence interval (CI) for HR crosses the null hypothesis (HR = 1), indicating a loss of significance. This dynamic effect may relate to a temporary immune response triggered by AL and challenges in restoring nutritional status, possibly due to immunological impairment from severe AL. Based on these findings, we can speculate that AL negatively affects survival during the first four years of follow-up. Hence, implementing stringent surveillance protocols and a structured rehabilitation strategy could help alleviate nutritional deficiencies and potential immunological effects, considerably impacting long-term survival. Therefore, AL should be carefully considered in oncologic follow-up during the first 48 months, as these patients likely require a closer monitoring protocol.

Four principal issues should be considered when analyzing our findings. First, centralization in high-volume centers has been found to notably enhance short-term outcomes, reduce 90-day mortality, and significantly reduce the postoperative AL risk [44], with this effect attributed to higher surgeon experience, multidisciplinary management, easier access to specialized facilities, earlier identification of issues, and lower “failure to rescue” rates. In this study, proper stratification by hospital volume was not feasible. Second, the severity of AL is a critical factor and may influence OS. Kamarajah et al. [14] noted that severe and non-severe ALs were both associated with shorter long-term survival (median 24.2 and 40.9 months, respectively, vs. 59.8 months in the no-leak group; *p* = 0.013), but in adjusted Cox regression analysis, only severe AL was associated with poor long-term survival (HR = 3.96, 95%CI 2.11–7.44; *p* < 0.001). However, a precise sub-analysis based on AL severity was not possible due to varying definitions (Supplementary Table S1) across studies. Third, none of the included studies clearly defined the timing of AL diagnosis or the efficacy of treatments (e.g., stents, vacuum therapy, clips, conservative management) concerning fistula closure. Therefore, the influence of caseload centralization, AL severity, and the timing of AL diagnosis and treatment on long-term survival necessitates prospective investigations. Stratification based on the pathological stage was not possible as it was not reported or reported in a heterogeneous way across the various studies.

Lastly, neoadjuvant chemotherapy (NAC) for gastric cancer remains clinically significant as it downstages tumors and enhances both progression-free and overall survival [45,46]. According to the European Society for Medical Oncology (ESMO) guidelines [47], perioperative chemotherapy is recommended for patients with resectable gastric cancer at stage IB or higher. While the association between NAC and postoperative complications continues to be investigated, the most current evidence indicates that patients receiving NAC experience similar rates of postoperative complications compared to those undergoing upfront surgery, suggesting no increased postoperative risk [48,49,50]. Further, it has been reported that NAC might have a prophylactic effect in patients who underwent gastrectomy and experienced postoperative complications. Specifically, Eto et al. in their 2018 retrospective single-center study, including 101 patients, concluded that NAC might abolish the poor prognosis induced by postoperative complications [51]. In their paper, the authors focused on postoperative complications (Clavien–Dindo > 1) but failed to report the exact number of AL and to perform a dedicated analysis on AL. Hence, the exact impact of NAC in patients who experienced AL after gastrectomy remains unsolved. In this study, only four papers reported heterogeneous and aggregate data for NAC and AL; therefore, a dedicated subgroup analysis was unfeasible. Additional research is needed to elucidate this relatively unexplored area.

The primary strength of our meta-analysis lies in its assessment of long-term survival differences between AL vs. no AL patients using HR and RMSTD. The RMSTD has gained considerable recognition in oncology, as it is a trustworthy, robust, and interpretable tool to assess the clinical survival benefit of a specific treatment over another [40]. By aligning with the area under the survival curves, it offers greater clarity compared to HR and RR, which can be misinterpreted due to their assumption of constant risk throughout follow-up. However, we acknowledge that this study has certain limitations, including potential publication bias and baseline heterogeneity among patients (i.e., demographics, comorbidities, nutritional status, etc.). Further, the definition of leak was heterogeneous across studies. In some studies, essential oncologic data such as staging, histology, grading, and the extent of lymphadenectomy were either absent or reported sporadically.

Additionally, our findings may not be universally applicable due to variations in epidemiology, genomic characteristics, anastomosis location and type (EJ versus GJ anastomosis), surgical approaches (open vs. hybrid), adjustments for early mortality (30 vs. 90 days), and perioperative management. However, even with a high variability in the baseline data, these parameters were reported in aggregated form or in an inconsistent way, and therefore, it was not possible to adjust for these confounding factors. Moreover, no details on the cause of death were available; therefore, the sole interpretation of OS can be misleading. Future studies would benefit from a more precise analysis of other survival indicators, such as cancer-specific and disease-free survival.

## 5. Conclusions

This study seems to suggest a clinical impact of AL on long-term OS after gastrectomy. Patients who experience AL seem to have a higher mortality hazard during the first four years of follow-up.

## Figures and Tables

**Table 1:** Demographic and clinical characteristics of patients undergoing gastric resection. Tumor location: upper (U), medium/body (M), lower (L), whole stomach (WS), other site (O); neoadjuvant therapy (NA), adjuvant therapy (A); histology type: differentiated (D), undifferentiated (UN), signet ring cell carcinoma (SRC), diffuse (DF), indeterminate (I), complete response (CR); American Joint Committee on Cancer (AJCC), Union for International Cancer Control (UICC), pathological stage (pS); American Society of Anesthesiologists risk listed from 1 to 4 (ASA); anastomotic leak (AL); hospital length of stay expressed in days (HLOS); without leak (WOL), with leak (WL); lymphadenectomy listed as D1/D1+/D2; surgical procedure listed as distal gastrectomy (DG) and total gastrectomy (TG), proximal (P), pylorus-preserving (PP), completion gastrectomy (CG); surgical approach listed as open (Op), hybrid (H), minimally invasive.

Author, Country, Year	Period	No. Patients	Age	M/F	BMI	Tumor Location	NA/A	Histology	pS 0-I	pS IIa	pS IIb	pSIII	pSIV	N0	N1	N2	N3	ASA	Surgical Procedure (DG-TG)	Lymphadenectomy (D1-D1+-D2)	Approach (Op-H- TMI)	Operating Time (In Min)	AL	HLOS	30d Mortality
Sierzega, Poland, 2010 [15]	1999–2004	690	63.7 (26–84)	458/232	nr	290U/103M 148L/149WS	0–196	nr	nr	nr	nr	nr	nr	202	185	128	175	nr	0/690	123/332/235	690/0/0	300 < 240 m; 390 ≥ 240 m	41	13/31	27
Yoo, South Korea, 2011 [23]	2000–2005	478	58 ± 10	326/152	nr	250UM/228L	Nr–363	172D/306UN	nr	nr	nr	nr	nr	177	173	70	58	nr	295/183	37/426/15	478/0/0	nr	32	nr	nr
Nagasako, Japan, 2012 [24]	1997–2008	400	63.4 (32–92)	278/122	22.2 (17.1–30.8)	48U/216M/107L/29ML	0–0	252D/148UN	nr	nr	nr	nr	nr	368	nr	nr	nr	nr	296/33 (44P/27PP	166/168/66 (D1/D1+/D2)	0/400/0	303.3 (152–865)	14	nr	nr
Kim, South Korea, 2015 [25]	2003–2012	3827	1722 < 60/ 2105 ≥ 60	2602/1225	2683 < 25 1045 ≥ 25 3	733U/1013M/ 1935L/81WS	nr	nr	2249	579		875	113	2298	482	369	671	3476 < 3 351 ≥ 3	2913/752 (162P)	nr	1460/2367/0	3616 ≤ 300 m; 211 > 300 m	72	15.8WL	0
Barchi, Brazil, 2019 [26]	2009–2017	258	62.3 (25–94)	180/78	23.8	170ML	44–123	107D/151UN	113			145		91	167			202 < 3/56 ≥ 3	0/208 (50CG)	90/168 (D0-D1/D2)	244/0/14	nr	15	11.6/36.7	11
Nagata, Japan, 2022 [27]	2012–2018	197	73.2 ± 10.2	135/62	nr	29U/82M/86L	0–nr	9D/75 SRC	nr	nr	nr	nr	nr	103	34	31	29	nr	136/61	nr	105/92/0	356.5	9	nr	0
Mittelstadt, Germany, 2022 [28]	2000–2018	356	65 ± 12	254/102	26.0 ± 4.3	nr	183–13	nr	88	75		92	80	nr	nr	nr	nr	15/211/125/5	0/100	nr	356/0/0	nr	22	nr	nr
D’Souza, New Zeland, 2023 [29]	2014–2022	77	65 (54–76)	47/30	nr	39ML/25 U	43–382	4 DF/2 I/4CR	31	23		22	1	47	30			8/29/36/4	25/52	22/42/0	70/7/0	nr	6	8	1%
He, China, 2023 [12]	2014–2021	3926	1394 < 60/ 2532 ≥ 60	2845/1081	789 <25/1031 ≥25 AND <30/106 ≥3	1146U/1127M 1456L/197O	184–nr	nr	1450	897		1532	47	1859	552	557	958	3335 < 3 591 ≥ 3	1967/1818 (141P)	nr	1493/2433/0	2095 < 180 m; 1831 ≥ 180 m	80	nr	nr
Ishida, Japan, 2023 [30]	1997–2018	1653	1288 < 75/ 365 ≥ 75	1113/540	nr	nr	nr	nr	nr	nr	nr	nr	nr	1191	461			nr	nr	nr	1069/571/13	nr	47	nr	nr

**Table 2:** The 60-month restricted mean survival time difference (RSMTD) for leak vs. no leak comparison across different time horizons for OS. SE: standard error; CI: confidence interval; mos: months.

Time Horizon	No. Trials	RMSTD (mos)	SE	95% CI	*p*-Value
12 months	10	−1.1	0.3	−1.6, −0.5	0.00008
24 months	10	−3.1	0.8	−4.7, −1.5	0.0002
36 months	9	−5.2	1.3	−7.8, −2.6	0.00008
48 months	9	−8.1	1.9	−11.8, −4.4	0.00001
60 months	8	−10.6	2.5	−15.4, −5.7	0.00001

**Table 3:** The 60-month hazard ratio (HR) analysis for leak vs. no leak. CI: confidence interval.

Time Horizon	HR (95% CI)
12 months	1.32 (1.11–1.58)
24 months	1.61 (1.34–1.92)
36 months	1.55 (1.27–1.91)
48 months	1.22 (1.02–1.53)
60 months	0.79 (0.59–1.10)

## Data Availability

Data generated at a central, large-scale facility are available upon request from the corresponding author.

## Supplementary Materials

The following supporting information can be downloaded at https://www.mdpi.com/article/10.3390/cancers17152471/s1: Figure S1: Risk of bias domains, Table S1: Definition of leak across studies.

## Appendix A

### Search Strategy

PubMed: (“gastric cancer” [MeSH Terms] OR “gastric neoplasm” [MeSH Terms] OR “gastric carcinoma” [MeSH Terms]) AND (“gastrectomy”) AND “leak” [MeSH Terms] or “anastomotic leak” OR “anastomosis leak” OR “anastomotic failure” OR “anastomosis failure” OR “anastomotic breakdown” OR “anastomosis breakdown”) AND (“survival” [MeSH Terms] OR “overall survival” [MeSH Terms].

Scopus: TITLE-ABS-KEY (“gastric cancer” OR “gastric neoplasm” OR “gastric carcinoma”) AND TITLE-ABS-KEY (“gastrectomy”) AND “leak” [MeSH Terms] or “anastomotic leak” or “anastomosis leak” OR “leakage” or “anastomotic failure” or “anastomosis failure” or “anastomotic breakdown” or “anastomosis breakdown” [MeSH Terms]) AND (“survival” [MeSH Terms] OR “overall survival” [MeSH Terms].

Google Scholar: “gastric cancer” OR “gastric neoplasm” OR “gastric carcinoma” AND “gastrectomy” AND “survival” OR “overall survival” AND (“leak” OR “leakage” OR “anastomotic leak” OR “anastomosis leak” OR “anastomotic failure” OR “anastomosis failure” OR “anastomotic breakdown” OR “anastomosis breakdown”).

Embase: (“gastric cancer” OR “gastric neoplasm” OR “gastric carcinoma”) AND (“gastrectomy”) AND (“survival” OR “overall survival”) AND (“leak” OR “leakage” OR “anastomotic leak” OR “anastomosis leak” OR “anastomotic failure” OR “anastomosis failure” OR “anastomotic breakdown” OR “anastomosis breakdown”).

Cochrane Central Library: (“gastric cancer” OR “gastric neoplasm” OR “gastric carcinoma”) AND (“gastrectomy”) AND (“survival” OR “overall survival”) AND (“leak” OR “leakage” OR “anastomotic leak” OR “anastomotic failure” OR “anastomosis failure” OR “anastomotic breakdown” OR “anastomosis breakdown”).
