# Supplementary figures and images for: Impact of Anastomotic Leak on Long-Term Survival After Gastrectomy: Results from an Individual Patient Data Meta-Analysis
Source: Cancers (Basel). 2025 Jul 25;17(15):2471. doi: 10.3390/cancers17152471 (PMC12346099; doi:10.3390/cancers17152471)

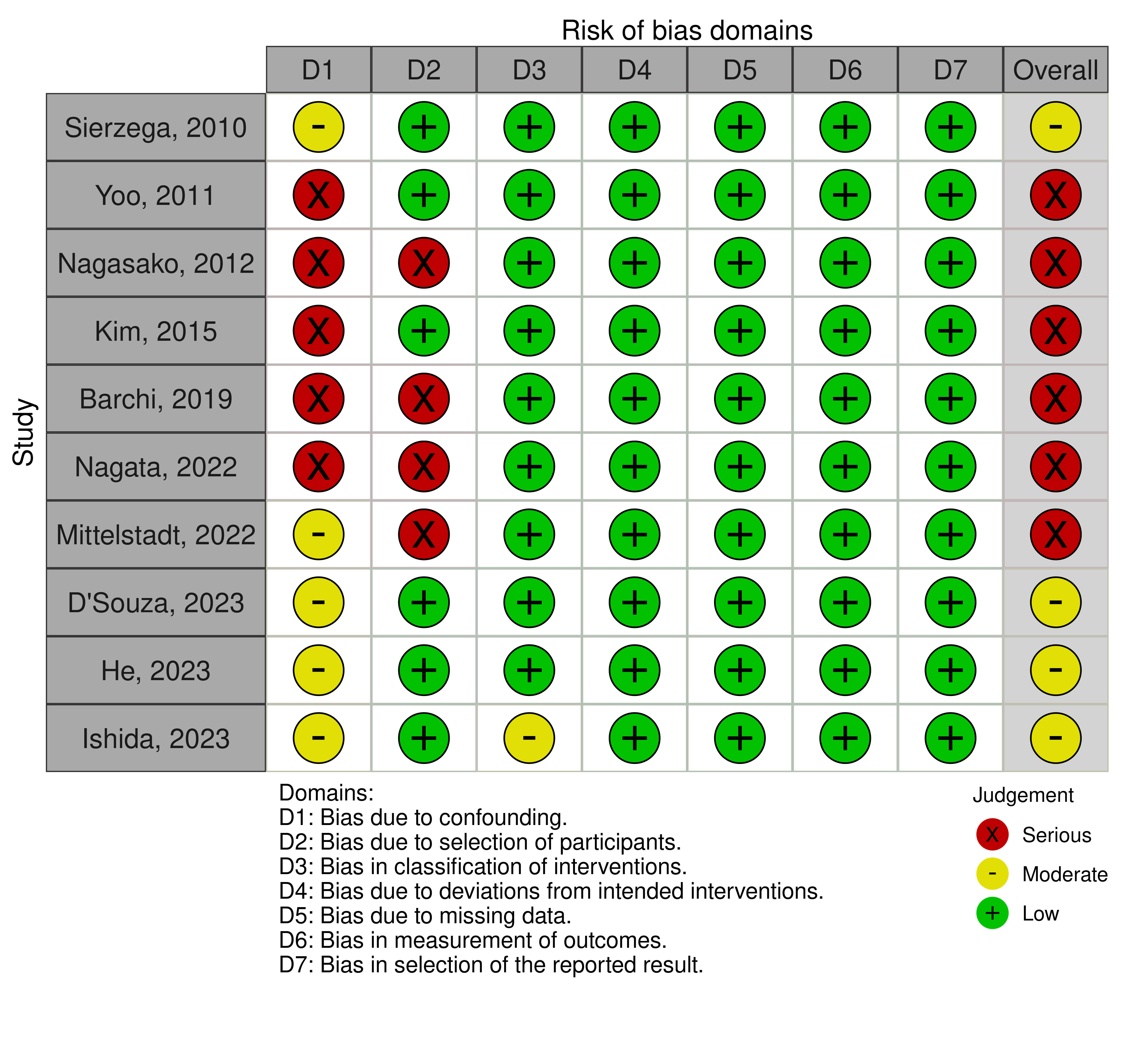

Supplement: Supplementary file 1 [file cancers-17-02471-s001.zip › cancers-3724781-Figure S1.jpg]
